# Sleep Disturbances and Patterns in Children With Neurodevelopmental Conditions

**DOI:** 10.3389/fped.2021.637770

**Published:** 2021-03-02

**Authors:** Elizabeth J. Halstead, Anna Joyce, Emma Sullivan, Carwyn Tywyn, Kyle Davies, Alexandra Jones, Dagmara Dimitriou

**Affiliations:** ^1^Sleep Education and Research Laboratory, Psychology & Human Development Department, University College London, Institute of Education, London, United Kingdom; ^2^Faculty of Humanities, Arts and Social Sciences, School of Psychotherapy & Psychology, Regent's University London, London, United Kingdom; ^3^Cerebra, For Brain Injured Children and Young People, Carmarthen, United Kingdom

**Keywords:** sleep, neurodevelopmental conditions, parent, child, medication, intervention, intellectual disabilities, autism

## Abstract

**Background:** Children with neurodevelopmental conditions (NDC) often experience sleep problems which are long-lasting and more complex than typically developing children. These sleep problems impact their families and there is little guidance for management specifically for sleep for families of children with neurodevelopmental conditions. The present study aims to use parental report to evaluate sleep disturbances and sleep patterns in a large sample of children with NDC. We aim to identify associations with age, diagnosis, and medication groups.

**Methods:** Data on 601 children aged between 2 and 17 years was analyzed from a UK non-profit service for sleep for families of children with NDC. Parents/carers completed the children's sleep habit questionnaire, a 7 day sleep diary, and information on child age, diagnosis, and medication. Parents also reported previous sleep management techniques they had tried.

**Results:** Overall, we found differences between age, diagnosis, and medication use groups for sleep disturbances and sleep diary parameters in these populations. Sensory conditions were associated with high night time waking duration. Parents reported their child's short sleep duration was the most common problem for them.

**Conclusions:** Key areas for further research are outlined including the long term considerations for parental presence at bedtime for sleep anxiety, melatonin use and efficacy, and consideration for interventions to reduce daytime fatigue in children aged 7–11 years old.

## Introduction

It has been established that children with Neurodevelopmental Conditions (NDC) often experience sleep disturbances which are long-lasting and more complex than typically developing children (1). Approximately 40 to 80% of children with NDC encounter sleep problems such as sleep onset delay, frequent nocturnal wakings, bed-wetting, and nightmares (2–4).

Only recently, sleep has begun to be examined and included in the characterization of several NDCs, including Autism Spectrum Disorders (ASD), Cerebral Palsy (CP), Down Syndrome (DS), Fetal Alcohol Syndrome (FASD), and Williams syndrome (WS) (5). As an example, sleep problems in WS have recently been added to the WS clinical management guidelines (6).

In children with ASD, sleep disturbances include sleep onset delay, frequent nocturnal wakings, bedtime resistance, and reduced total sleep time (7–9). In CP, sleep disturbances include insomnia, difficulty with sleep onset, night wakings, daytime fatigue, nightmares, and sleep apnoea (10, 11). Sleep disturbances are common in children with DS occurring in ~31–54% of the population (12–14). Children with DS tend to experience sleep-disordered breathing due to the craniofacial and medical characteristics of the disorder often causing airway obstruction (e.g., small airway and weak pharynx) (10). It thus appears that children with different developmental disorders often experience sleep problems which are specific to their conditions, hence, may require different clinical intervention or management.

Insufficient sleep has been found to adversely impact developing children's cognitive functioning including memory, attention, and learning which often leads to the exacerbation of challenging behavior and family stress (1, 15). These findings are of particular concern in children whose neurodevelopment is already following atypical or delayed cognitive developmental pathway (16–20).

Also, several studies have shown that children with NDC who were diagnosed with sleep disorders had disturbed behavioral functioning and scored higher on aggression, screaming, temper tantrums, and non-compliance than children with NDC without sleep disorders (21). In addition, Goldman et al. (22) found hyperactivity, social interaction delay, and mood disorder symptoms were magnified in children with ASD who experienced sleep problems. Children with ADHD are often reported to have sleep problems, which in turn can further exacerbate ADHD symptoms, for example, strong correlations have been found between sleep problems in children diagnosed with ADHD and increased daytime emotional dysfunctional behavior (23). The impact of sleep problems increasing behaviors associated with ADHD is further supported by Knight and Dimitriou (24), who reported that typically developing (TD) children experiencing sleep problems scored higher on an ADHD Conners Rating Scale (25), suggesting that severe sleep problems can result in ADHD-like behaviors in TD children (24).

In addition, anxiety has been found to impact sleep in children with NDC and there is a bidirectional association between anxiety and sleep present in the literature. Children with ASD are more likely to experience more sleep problems if they have co-occurring psychiatric issues such as anxiety or depression (16). Furthermore, children with DS and CP have been identified as suffering from sleep anxiety, magnifying their bedtime resistance, and increasing their sleep onset time (16).

The complex nature of sleep and its impact on children with NDC suggests that provisions ought to be extended to families to effectively manage sleep disturbances which is essential for family functioning. Evidence has suggested that maternal mental health issues, family disruption, and deficient parent-child relationships are associated with children's sleep disorders (26). Gallagher et al. (27) found that 72% of caregivers to children with intellectual and developmental disabilities (IDD) considered themselves to be sleep deficient. This was compared to 22% from the control group of parents of TD children (27). Although limited evidence suggests that sleep hygiene practices can reduce the anxiety many children with IDD or ASD experience, thus promoting a more efficient sleep (28), a lack of sleep education specific to parents of children with IDD has been identified (29). Feasibly, this is due to the difficulty of tailoring suitable sleep education and hygiene practices to the specific needs and individual circumstances of the children and their families (29).

The precise profiling of sleep patterns and disorders in NDC is still in its infancy (10, 30, 31). Since sleep intertwines with environmental and biological factors, considerations for age, diagnosis, and co-occurring conditions along with the presence of medication ought to be examined to develop successful sleep management in this population. The National Sleep Foundation recommendations for sleep duration by age are based on typically developing children, however, these recommendations do not consider the needs of NDC populations (32). Previous research has suggested that children with NDC have different sleep profiles as early as preschool age (33). Following these recommendations can cause stress to families, particularly if their child seemingly does not “fit” into these recommended sleep duration hours, or if the family experience difficulties co-ordinating sleep schedules of TD children and their siblings with NDC. Many of the previous studies focusing on sleep profiles in NDC populations only focused on differences between a limited number of conditions, families whose children have rare or complex disorders are limited in evidence they can refer to for information relating to sleep disturbances [see review (34)]. In the context of providing clinical services for families of children with NDC for their sleep, it is important to have an overview of typical sleep disturbances and patterns seen in the NDC population and to consider associated factors that may directly impact the clinical advice. In this study, we specifically focus on age, diagnosis group, and medication of children. In addition, there is limited evidence on the impact of medication, and specifically sleep medication on sleep in these populations (35).

The present study aims to use parental report to evaluate sleep disturbances and sleep patterns in a large sample of children with NDC. We aim to identify associations with age, diagnosis, and medication groups. In addition, parental report of children's primary sleep problem and endeavored sleep management techniques (parental intervention) will be reported and discussed. Together, this analysis provides insight into the sleep disturbances experienced by these populations and their families, clinical considerations are discussed.

## Materials and Methods

### Recruitment

In collaboration with a UK non-profit organization, Cerebra, that supports families of children with NDC, data were collected from families who approached their sleep service from 2016 to 2019. The sleep service provided by the non-profit organization is dedicated to supporting families of children and young people with NDCs to manage their sleep problems. Ethical approval was obtained from a UK institutional review board to analyze the anonymized data for research purposes.

### Participants

Data were analyzed for 601 children with NDC, aged between 2 and 17 years (*M* = 7.45, *SD* = 3.52) whose parents/carers contacted the service regarding a sleep problem. All data were collected via parental/carer reporting. Chronological age was categorized according to the UK Department for Education national curriculum key stage (KS) age groups (see Table 1) to establish disparities in ages in the context of educational settings (e.g., nursery, school) and learning.

**Table 1:** Number of participants in each age group.

**Ages**	***n***	**%**
2–3 years	38	6.3
3–5 years (KS0)	144	24.0
5–7 years (KS1)	124	20.6
7–11 years (KS2)	200	33.3
11–14 years (KS3)	60	10.0
14–16 years (KS4)	25	4.2
>16 years (KS5)	10	1.6
Total	601	100

### Measures

Parents/carers completed an intake form which included demographic data, the Child Sleep Habits Questionnaire, and information on behavioral sleep management techniques the parents had endeavored prior to contacting the sleep service.

#### Demographic

Demographic information gathered and available included child's age, primary diagnosis, and co-occurring conditions, and current medications. Parents were also asked to report the sleep intervention/s (e.g., parental presence) they had tried prior to contacting the sleep service.

#### Child Sleep Diary

Parents completed a sleep diary for their child's sleep in the last week. The sleep diary included the following sleep parameters: Bedtime, falling asleep time, duration of night wakings (in minutes), final wake up time, and estimated assumed total sleep time. These parameters were reported, and the weekly means were calculated to the nearest hour.

#### Child Sleep Habits Questionnaire

Parents completed the Child Sleep Habits Questionnaire (CSHQ), a 33-item parent-report questionnaire covering characteristics of sleep disorders in children. Parents report on a three-point scale (rarely, sometimes, and usually) for the occurrence of sleep characteristics such as bedtime struggles, snoring, and night wakings over the past week, and whether they consider that sleep characteristic a problem. Items were rated on a binary scale from 0 (no problem) to 1 (problem) and a composite problem subscale was computed from adding all the problem scores. The CSHQ yields a total sleep disturbance score as well as scores on eight subscales: bedtime resistance, sleep onset delay, sleep duration, sleep anxiety, night wakings, parasomnias, sleep disordered breathing, and daytime sleepiness. The CSHQ has satisfactory test–retest reliability for both normal and clinical populations (27, 36) and has been used in children aged 2–18 years (37).

### Analysis Approach

Data were analyzed using the IBM SPSS version 25. Kurtosis skewness, Shapiro-Wilk test of normality and visual inspection of histograms suggested non-normal distribution of data for the majority of variables in this clinical sample; however, given the central limit theorem and our large sample size, parametric tests are used throughout for improved statistical power (38).

Sleep disturbances are categorized using the eight subscales of the CSHQ and sleep parameters are reported from the sleep diary. For sleep subscales and parameters, analyses were conducted with demographic variables age, diagnosis, and medication. Associations between chronological age and each sleep subscale or parameter were conducted using Pearson's correlations. Key Stage age groups were compared using ANOVA with 1,000 bootstrapped samples for robust measures. When initial tests were significant, pairwise comparisons were conducted with Bonferroni correction for multiple comparisons. Where assumptions of homogeneity were violated, Welch's F statistic is reported along with pairwise comparisons with Games-Howell correction for multiple comparisons.

Group comparisons based on children's primary diagnosis and primary medication were made on all sleep subscales and parameters. Power calculation for an *F*-test in G^*^Power yielded a sample size of 297 based on ANCOVA with up to 14 groups (the total number of primary medication groups) and one covariate, with a medium effect size, 80% power and an alpha level of 0.05. Groups smaller than five subjects were excluded. Sensory disorders have been associated previously with sleep disturbances (40); therefore, a dichotomous variable was created to include children reported to have a primary or secondary sensory disorder vs. no sensory disorder. Melatonin use was also dichotomized since this is a medication used to treat sleep problems, thus was expected to be of interest. For the group taking melatonin (*n* = 112) mean age was 7.57 years (SD = 3.65), and for the group not taking melatonin (*n* = 485) mean age was 7.43 (SD = 3.51), a *t*-test found no significant differences in age between the two groups [*t*_(595)_ = 0.38, *p* = 0.701]. All group comparisons for each sleep subscale and parameter were made using ANCOVA controlling for age with 1,000 bootstrapped samples. For *post-hoc* comparisons, diagnostic groups were each compared to one another; medication groups were compared to the no medication group. The Bonferroni method was used to correct for multiple comparisons.

Descriptive statistics for the sleep problem items of the CSHQ are ranked within diagnostic groups and presented. Of the parents who had indicated a type of intervention they had tried with their child, descriptive statistics are presented. *T*-tests were conducted to compare a dichotomized variable (if the child had parental intervention vs. no parental intervention), and the type of intervention (e.g., parental presence vs. no parental presence intervention) with each CSHQ subscale.

### Missing Data

Due to unanswered questions on the CSHQ, subscale scores could not be calculated for all variables for every participant, if one item or more on the subscale was missing, then the subscale was not calculated and that participant was excluded from the relevant analysis. For reporting of final *ns* for participants see Tables 4–6.

## Results

The following results present significant findings for: 1. Each sleep disturbance as measured by the CSHQ; 2. Sleep parameters measured by the parental sleep diary report; 3. Parental problems as measured by the CSHQ; and 4. Parental interventions used. For sections Introduction and Materials and Methods, associations of age, diagnostic groups, and medication are reported. Primary diagnosis and co-occurring conditions are reported in Table 2, ASD was the most prevalent condition, with 339 children diagnosed with ASD across all age groups. Overall, 221 children had at least one other condition co-occurring in addition to their primary diagnosis. Medication use as primary medication is reported in Table 3; 371 children were not on any medication. Overall, 18.60% of the children (*n* = *112)* were reported as taking melatonin for their sleep problem.

**Table 2:** Number of participants with each primary diagnosis and co-occurring condition.

	**Primary diagnosis**		**Co-occurring condition**		**Total**	
	***n***	**%**	***n***	**%**	***n***	**%**
ASD	339	56.4	6	1.0	345	57.4
LD/Dyspraxia/Dyslexia	30	5.0	80	13.3	110	18.3
ADHD	51	8.5	54	9.0	105	17.5
Brain Injury^*^	34	5.7	21	3.5	55	9.2
SEMH^**^	7	1.1	45	7.5	52	8.7
Physical condition^***^	2	0.3	28	4.7	30	5.0
Other ID^****^	31	5.2	1	0.2	32	5.3
Sensory disorders^*****^	9	1.5	60	10.0	69	11.5
Cerebral Palsy	14	2.3	–	–	14	2.3
Down's syndrome	18	3.0	–	–	18	3.0
Epilepsy	14	2.3	–	–	14	2.3
No disability specified	52	8.7	21	3.5	73	12.1
Total	601	100	316	52.7	–	–

*ASD, Autism Spectrum Disorder; LD, Learning Difficulty; ADHD, Attention Deficit Hyperactivity Disorder; SEMH, Social Emotional & Mental Health; please note this term is used in the UK, SEND, Special Education Needs and Disabilities, Code of Practice (39); ID, Intellectual Disabilities*.

* *Examples include: Cerebral Infraction, Hypoxic-ischemic encephalopathy*,

** *Examples include: Anxiety, Pathological Demand Avoidance*,

*** *Examples include: postural tachycardia syndrome, high blood pressure*,

**** *Examples include: Rett Syndrome, Angelman Syndrome*,

***** *Examples include: auditory/sensory processing disorders*.

**Table 3:** Number of participants on all medication types as primary medication.

**Primary medication of participants**	***n***	**%**
Stimulant medication	32	5.3
Seizure control medication	23	3.8
Allergies/Eczema	40	6.7
Gastrointestinal	34	5.7
Genitourinary	10	1.7
Respiratory	16	2.7
Musculoskeletal	3	0.5
Psychiatric	36	6.0
Blood/Lymphatic	7	1.2
Painkiller	8	1.3
Other (antibiotics/homeopathy)	13	2.2
Endocrine	6	1.0
Cardiovascular	2	0.3
No medication	371	61.6
Total	601	100

Means, standard deviations, and statistical analysis results for CSHQ and parameters according to age group, primary diagnosis, primary medication, and melatonin use are reported in Tables 4–7, retrospectively. Please refer to the Supplementary Material to contextualize this cohort with reference to a typically developing cohort and a clinical sleep disorders population previously reported by Owens et al. (36, 41).

**Table 4:** Means and standard deviations for key stage groups on sleep disturbances and parameter.

**Key Stage Group**	**2–3 years** **(*****n*** **=** **10–34)**		**KS0 (3–5 years)** **(*****n*** **=** **55–108)**		**KS1 (5–7 years)** **(*****n*** **=** **57–108)**		**KS2 (7–11 years)** **(*****n*** **=** **101–166)**		**KS3 (11–14 years)** **(*****n*** **=** **27–51)**		**KS4 (14–16 years)** **(*****n*** **=** **7–15)**		**KS5 (16** **+** **years)** **(*****n*** **=** **3–9)**		**Correlations**		**ANOVA** **main effects**		
	***M***	***SD***	***M***	***SD***	***M***	***SD***	***M***	***SD***	***M***	***SD***	***M***	***SD***	***M***	***SD***	***r***	***p***	***F***	***p***	**ES**
**Sleep disturbance**																			
Bedtime Resistance	11.33	3.15	11.73	3.7	12.3	3.27	12.31	3.43	9.89	3.59	8.8	2.62	8.57	2.15	−0.15	0.008^*^	4.46	<0.001	0.08
Sleep Onset Delay	2.42	0.72	2.34	0.79	2.49	0.75	2.66	0.66	2.5	0.7	2.55	0.52	2.71	0.76	0.15	0.002^*^	–	–	–
Sleep Duration	7.73	2.37	7.25	1.79	7.04	1.67	7.11	1.72	7.14	1.75	7.08	1.83	7.29	1.25	−0.03	0.541	–	–	–
Sleep Anxiety	7.8	2.37	8.09	2.74	9.03	2.23	9.12	2.44	7.39	3.03	6.09	2.66	7.14	2.19	0.1	0.099	4.55	0.001	0.07
Night Waking	7.32	1.6	6.81	1.61	6.24	2.1	6.19	2.03	5.94	2.05	5.36	1.75	5.75	1.71	−0.20	<0.001^*^	2.92	0.022	0.03
Parasomnias	10.5	2.46	12.09	3.59	11.33	2.79	11.08	2.69	10.4	2.74	8.7	1.7	10.17	1.6	−0.21	0.001^*^	4.14	0.003	0.07
SDB	4	1.38	4.26	1.63	4.03	1.52	3.98	1.54	3.87	1.36	3.14	0.38	3.43	0.53	0.11	0.054	–	–	–
Daytime Sleepiness	13.74	3.97	12.95	2.83	13.87	4.14	15.75	3.93	15.48	4.25	16.88	2.75	17.5	3.08	0.31	<0.001^*^	7.27	<0.001	0.11
**Sleep parameter**																			
Bedtime	20:16	1:12	19:46	1:05	19:51	0:53	20:36	1:11	21:05	0:56	21:51	1:39	21:20	1:31	0.42	<0.001^*^	15.22	<0.001	0.16
Falling asleep time	21:16	1:22	20:51	1:32	21:33	1:31	22:13	1:26	22:34	1:28	23:08	2:26	22:20	1:31	0.39	<0.001^*^	13.47	<0.001	0.15
Morning wake time	7:07	1:29	6:47	1:15	6:47	1:15	7:00	1.06	7:13	1:21	7:00	1:09	8:00	1:43	−0.09	0.088	–	–	–
Night Waking	74.27	55.22	86.35	63.97	51.85	46.28	54.65	66.51	61.52	61.23	55.27	103.14	150	114.9	0.1	0.034^*^	3.61	0.008	0.08
Sleep duration	8.68	1.51	8.83	1.81	8.65	1.84	8.11	1.98	8.14	2.17	7.07	3.37	6.78	3.63	−0.21	<0.001^*^	2.74	0.02	0.02

*NB: All correlations reported for association between age and sleep disturbance/parameter*.

* *significant at the 0.05 level*.

*All significant ANOVA effects reported for age group*.

*ES, Effect sizes for one-way ANOVAs partial η^2^and Welch's F ratio adjusted omega squared (ω^2^)*.

*SDB, sleep disordered breathing*.

**Table 5:** Means and standard deviations for primary diagnostic groups on sleep disturbances and parameters.

**Diagnostic Group**	**ASD** **(*****n*** ***=*** **149–272)**		**LD/dyspraxia** **dyslexia** **(*****n*** ***=*** **10–25)**		**ADHD** **(*****n*** ***=*** **25–44)**		**Brain injury** **(*****n*** ***=*** **18–30)**		**SEMH** **(*****n*** ***=*** **4–7)**		**Physical condition** **(*****n*** ***=*** **2)**		**ID** **(*****n*** ***=*** **11–28)**	
	***M***	***SD***	***M***	***SD***	***M***	***SD***	***M***	***SD***	***M***	***SD***	***M***	***SD***	***M***	***SD***
**Sleep disturbance**														
Bedtime Resistance	11.87	3.55	11.1	3.51	10.8	3.24	12	3.58	13	4.12	11	4.24	12.25	4.2
Sleep Onset Delay	2.57	0.68	2.38	0.72	2.58	0.69	2.43	0.79	3	0	3	0	2.11	0.9
Sleep Duration	7.03	1.8	7.79	1.12	7.47	1.63	7.46	1.63	8	1.22	8.5	0.71	7	1.56
Sleep Anxiety	8.62	2.51	7.67	2.5	8.65	2.61	8.41	3.19	9.6	2.88	7.5	3.54	7.82	2.96
Night Waking	6.3	1.93	6.57	1.6	6.61	2.03	5.87	2.12	5.2	2.05	6	1.41	7.07	2.09
Parasomnias	11.06	3.01	12	2.93	11.16	2.78	10.39	1.85	10.2	1.79	10.5	0.71	11.58	4.14
SDB	3.87	1.34	4	1.11	4.07	1.62	4.36	1.81	4.2	1.64	5.5	3.54	4.6	2.44
Daytime Sleepiness	14.89	3.89	14.08	4.66	13.03	3.65	14.78	3.92	15.5	2.89	20	5.66	13.69	3.59
**Sleep parameter**														
Bedtime	20:30	1:16	20:15	1:04	20:07	0:53	20:00	1:15	21:00	1:09	20:00	0:00	20:06	1:08
Falling asleep time	21:57	1:35	21:22	1:33	22:09	1:39	20:50	1:50	23:10	0:58	22:30	0:42	21:34	1:30
Morning wake time	6:59	1:20	6:45	0:56	6:53	0:59	6:36	1.18	7:08	0:41	7:00	0:00	6:25	1:10
Night Waking	67.68	65.83	69.55	46.21	63.21	71.95	67.27	55.37	144	138.13	90	42.43	60.71	53.13
Sleep duration	8.2	2.05	8.36	2.06	7.98	2.29	8.66	1.8	7	1.67	7	0	8.57	1.85
**Diagnostic Group**	**Sensory disorders** **(*****n=*** **4–7)**		**Cerebral palsy** **(*****n=*** **2–13)**		**Down's syndrome** **(*****n=*** **6–15)**		**Epilepsy** **(*****n=*** **5–11)**		**No disability specified** **(*****n=*** **25–41)**		**ANCOVA** **main effects**			
	***M***	***SD***	***M***	***SD***	***M***	***SD***	***M***	***SD***	***M***	***SD***	***F***	***p***	**Par** ***η*****2**	
**Sleep disturbance**														
Bedtime Resistance	10	2.28	9	1.41	10.43	2.88	10.6	3.65	12.77	3.64	–	–	–	
Sleep Onset Delay	2.5	0.84	2.27	0.9	1.93	0.92	2.55	0.69	2.63	0.69	1.96	0.045	0.05	
Sleep Duration	5.83	1.83	7.14	1.07	7.58	1.56	6.83	1.33	7.27	1.55	–	–	–	
Sleep Anxiety	7.2	1.79	7.71	3.2	7.83	3.37	6.33	3.14	9.42	2.28	–	–	–	
Night Waking	5.8	1.79	6.2	1.3	6.87	1.13	7.17	2.23	6.33	2.2	–	–	–	
Parasomnias	12.8	2.77	12.5	0.71	10.13	0.99	11.4	4.34	11.52	3.15	–	–	–	
SDB	3.4	0.89	3	0	5	1.67	3.17	0.41	4.36	1.55	–	–	–	
Daytime Sleepiness	17.67	3.98	15.22	3.49	11.88	2.53	10.6	1.67	16	3.94	4.04	<0.001	0.11	
**Sleep parameter**														
Bedtime	19:51	1:04	19:55	1:15	19:24	0:49	20:18	1:29	20:09	1:12	2.49	0.006	0.05	
Falling asleep time	21:25	1:43	21:09	1:27	20:04	1:01	21:36	1:53	21:56	1:33	3.55	<0.001	0.07	
Morning wake time	7:08	0:22	6:46	1:52	6:38	0:50	7:48	1:37	7:19	1:17	–	–	–	
Night Waking	105.5	89.22	39.38	33.78	51.92	49.23	72.5	62.5	50.91	60.85	–	–	–	
Sleep duration	8.86	1.57	9	1.87	10.07	0.83	9.3	1.7	8.8	2.14	–	–	–	

*NB: All significant ANCOVA effects reported for diagnostic group controlling for age*.

*Par η^2^ = Parital η^2^*.

**Table 6:** Means and standard deviations for primary medication group on sleep disturbances and parameters.

**Medication group**	**Stimulants** **(*****n*** ***=*** **15–27)**		**Seizure control** **(*****n*** ***=*** **8–20)**		**Allergies/** **Eczema** **(*****n*** ***=*** **16–37)**		**Gastrointestinal** **(*****n*** ***=*** **16–32)**		**Genitourinary** **(*****n*** ***=*** **2–9)**		**Cardiovascular** **(*****n*** ***=*** **1–2)**		**Respiratory** **(*****n*** ***=*** **8–13)**		**Musculoskeletal** **(*****n*** ***=*** **1–3)**	
	***M***	***SD***	***M***	***SD***	***M***	***SD***	***M***	***SD***	***M***	***SD***	***M***	***SD***	***M***	***SD***	***M***	***SD***
**Sleep disturbance**																
Bedtime Resistance	10	3.31	9.21	2.97	10.06	2.69	11.89	3.1	8	0	17	–	10.85	3.46	10	5.66
Sleep Onset Delay	2.61	0.66	2.52	0.68	2.42	0.88	2.39	0.78	2.2	1.1	2	–	2.62	0.65	2	1.41
Sleep Duration	7.8	1.61	7.5	1.63	7.09	1.8	7.38	1.36	7.8	0.45	9	–	7.23	1.09	7	1.41
Sleep Anxiety	7.87	2.95	6.5	2.47	7.89	2.17	10.5	1.93	9	2.45	12	–	8.5	1.9	7	4.24
Night Waking	6.6	1.96	6.5	1.87	5.58	1.89	7.1	1.65	7	1.77	7	–	7.25	1.28	6.5	0.71
Parasomnias	11.13	2.42	9.38	1.69	10.94	2.41	11.65	3.08	12	0.71	9	–	10.13	2.17	18	–
SDB	4	1.7	3.8	1.47	3.94	1.3	4.43	1.72	6	1.9	3	–	4.67	1.58	3	–
Daytime Sleepiness	14.07	4.53	12.89	4.48	14.67	3.4	14.39	3.53	17.25	4.99	20	–	16.5	3.66	13	–
**Sleep parameter**																
Bedtime	20:02	1:01	20:10	1:17	20:20	0:50	20:02	1:17	21:00	1:24	22:00	0:00	20:16	1:16	20:00	1:00
Falling asleep time	21:28	1:52	21:13	1:51	22:03	1:40	21:17	1:29	22:00	1:46	22:30	0:42	21:05	1:30	20:40	0:34
Morning wake time	6:31	1:15	7:06	1:42	7:01	0:51	6:56	1.13	7:07	0:59	7:30	0:42	7:16	0:47	5:40	1:09
Night Waking	56	54.77	52.78	47.53	65.71	64.4	52.88	54.67	84	53.67	17.5	17.68	63.13	66.71	70	45.83
Sleep duration	8.41	2.33	9	1.97	8.14	1.9	8.5	2.24	8.11	1.76	9	1.41	9.08	1.55	8.33	1.53
	**Psychiatric** **(*****n=*** **19–26)**		**Blood/lymphatic** **(*****n=*** **1–6)**		**Painkiller** **(*****n=*** **1–6)**		**Endocrine** **(*****n=*** **2–6)**		**Other (e.g., antibiotics,** **homeopathy)** **(*****n=*** **6–11)**		**No medication** **(*****n=*** **162–295)**		**ANCOVA** **main effects**			
	***M***	***SD***	***M***	***SD***	***M***	***SD***	***M***	***SD***	***M***	***SD***	***M***	***SD***	***F***	***p***	**Par** ***η***^**2**^	
	11.95	4.05	12	4.9	18	–	12	4.58	11.86	4.45	12.19	3.42	2.58	0.014	0.06	
	2.54	0.59	2.17	0.98	3	0	3	0	2.36	0.81	2.54	0.72	–	–	–	
	6.83	1.75	6.75	1.5	8.5	0.71	8.25	0.96	8	1.63	7.05	1.76	–	–	–	
	8.75	3.19	7	4.24	10.5	2.12	9	3	8.13	2.85	8.57	2.55	2.76	0.006	0.07	
	6.3	2.2	5	2.31	7.5	2.12	5.5	3.54	7.45	1.69	6.22	1.96	2.3	0.021	0.05	
	11.05	3.96	13	7.07	15	5.66	14	0	12.25	3.24	11.03	2.84	–	–	–	
	4.5	1.74	3	–	6	4.24	5	2	3	0	3.86	1.36	3.49	0.001	0.09	
	14.35	3.36	20.5	0.71	20.5	3.54	15.67	6.51	13.25	2.92	14.63	3.95	–	–	–	
	20:16	1:20	20:15	0:57	19:50	1:10	20:00	1:24	20:40	1:24	20:21	1:15	–	–	–	
	22:07	1:47	20:45	0:30	21:48	1:28	21:45	2:45	22:06	2:07	21:50	1:35	4.56	0.033	0.01	
	6:31	1:19	7:45	1:30	7:12	0:50	6:45	0:57	6:40	1:07	6:59	1:20	–	–	–	
	71.74	68.99	65	60.5	93	69.07	67.5	60.62	51.67	38.17	68.1	67.96	–	–	–	
	8.19	3.06	9.5	3.08	7.17	2.99	9.33	2.5	7.67	1.23	8.34	1.89	–	–	–	

*NB: All significant ANCOVA effects reported for medication group controlling for age*.

*Par η^2^ = Parital η^2^*.

**Table 7:** Means and standard deviations on sleep disturbances and sleep parameters for children taking melatonin vs. not taking melatonin.

**Group**	**Melatonin**		**No Melatonin**		**T-test main effects**		
	***M***	***SD***	***M***	***SD***	***t***	***p***	**Cohen's d**
**Sleep disturbance**							
Bedtime Resistance	11.29	3.65	11.81	3.49	–	–	–
Sleep Onset Delay	2.38	0.78	2.55	0.70	–	–	–
Sleep Duration	7.37	1.62	7.12	1.70	–	–	–
Sleep Anxiety	8.49	2.82	8.51	2.59	–	–	–
Night Waking	6.53	2.03	6.30	1.93	–	–	–
Parasomnias	10.58	2.63	11.23	2.96	–	–	–
SDB	3.83	1.44	4.05	1.52	–	–	–
Daytime Sleepiness	14.75	3.85	14.64	3.95	–	–	–
**Sleep parameter**							
Bedtime	20:20	1:12	20:19	1:13	–	–	–
Falling asleep time	21:25	1:38	21:50	1:37	−2.16	0.032	0.26
Morning wake time	06:42	1:20	07:00	1:16	−1.98	0.049	0.23
Night Waking	80.15	68.79	61.81	62.25	2.11	0.037	0.28
Sleep duration	8.24	2.29	8.41	1.97	–	–	–

*NB: All significant t-test effects reported for melatonin group*.

*SDB, sleep disordered breathing*.

### Sleep Disturbances

#### Bedtime Resistance

Bedtime resistance significantly decreased with age. There was a significant difference between age groups on the bedtime resistance subscale. Pairwise comparisons indicated children aged 5–7 (KS1) had significantly higher bedtime resistance scores than older children aged 11–14 (KS3) (*p* = 0.043). Children aged 7–11 (KS2) had a significantly higher bedtime resistance score than children aged 11–14 (KS3) (*p* = 0.021) and children aged 14–16 (KS4) (*p* = 0.042).

No significant differences were found between diagnosis or sensory diagnosis groups for bedtime resistance. There was a significant difference between medication groups. Comparisons with the no medication group showed that children taking seizure control medication had lower scores for bedtime resistance (*p* = 0.014). There was no effect of melatonin use on bedtime resistance.

#### Parasomnias

Parasomnias significantly decreased with age. Analysis revealed a significant difference between age groups on the parasomnia subscale. *Post-hoc* tests indicated children aged 3–5 years (KS0), 5–7 years (KS1), and 7–11 years (KS2) all had a higher parasomnia score than older children aged 14–16 years (KS4) (*p* = 0.001; *p* = 0.011; *p* = 0.019), respectively. No significant differences were found between diagnosis, sensory, medication, or melatonin groups for parasomnias.

#### Sleep Onset Delay

Problems with sleep onset delay significantly increased with age however there were no significant differences between age groups. There was a significant difference between diagnosis groups on the sleep onset delay subscale; however, no significant group differences emerged after controlling for multiple comparisons. There was no significant effect of sensory diagnosis, medication group, or melatonin group on sleep onset delay.

#### Daytime Sleepiness

Daytime sleepiness significantly increased with age. Findings indicated a significant difference between age groups on the daytime sleepiness subscale. *Post-hoc* comparisons revealed children aged 7–11 years (KS2) had a higher daytime sleepiness score than children aged 3–5 years (KS0) (*p* < 0.001) and 5–7 years (KS1) (*p* = 0.040). Children aged 14–16 years (KS4) had a higher daytime sleepiness score than children aged 3–5 years (KS0) (*p* = 0.045).

There was a significant difference between primary diagnosis groups on the daytime sleepiness subscale. *Post-hoc* tests showed that children with primary diagnosed sensory disorders (*p* = 0.045), cerebral palsy (*p* = 0.045), and unspecified conditions (*p* = 0.045) experienced higher daytime sleepiness score relative to children with ADHD. Children with ASD (*p* = 0.045), sensory disorders (*p* = 0.045), cerebral palsy (*p* = 0.045), and unspecified conditions (*p* = 0.045) had increased daytime sleepiness relative to children with epilepsy. Children with sensory disorders had higher daytime sleepiness score in comparison to children with DS (*p* = 0.045) whilst children with DS had higher scores than children with unspecified conditions (*p* = 0.045). Comparison of children with vs. without a sensory diagnosis showed no significant effect on daytime sleepiness. No significant differences were found between medication or melatonin groups and daytime sleepiness.

#### Sleep Anxiety

Correlation analysis revealed no significant association of sleep anxiety with age. Findings revealed a significant difference between age groups on the sleep anxiety subscale. *Post-hoc* tests indicated children aged between 7 and 11 years (KS2) had a higher sleep anxiety score than older children aged 14–16 years (KS4) (*p* = 0.041).

In addition, there was a significant positive association between CSHQ sleep onset delay subscale score and CSHQ sleep anxiety subscale score [*r*(287) = 0.12, *p* = 0.038], therefore, as sleep onset delay increased, sleep anxiety also increased.

No significant differences were found between diagnosis or sensory groups for sleep anxiety. There was a significant difference between primary medication taken and the sleep anxiety subscale. Comparisons with the no medication group showed that children taking seizure control medication had lower levels of sleep anxiety (*p* = 0.024), whilst children taking gastrointestinal medication had higher levels of anxiety (*p* = 0.008). There was no effect of melatonin on sleep anxiety.

#### Sleep Disordered Breathing

No significant correlation was found between age and sleep disordered breathing. In addition, no associations were found between age, diagnosis, or sensory groups for sleep disordered breathing.

There was a significant difference between primary medication taken and the sleep-disordered breathing subscale. Comparisons with the no medication group revealed that children on genitourinary medications had a significantly higher sleep disordered breathing score (*p* = 0.008), whilst children on other medication (e.g., antibiotics/homeopathy) had a lower score (*p* = 0.008). There was no effect of melatonin on sleep disordered breathing.

#### Sleep Duration

No significant correlation was found between age and sleep duration and no significant age group differences were found for the sleep duration subscale. Furthermore, no differences were found for diagnosis, sensory, medication, or melatonin groups for the sleep duration subscale.

#### Night Waking

Correlation analysis revealed the problems with night waking significantly decreased with age. There was a significant difference between age groups on the night wakings subscale; however, after correcting for multiple comparisons, no significant group differences were found. No significant differences were found between diagnosis or sensory groups for night waking.

There was a significant difference between medication groups for night wakings. Children taking other medication (e.g., antibiotics/homeopathy) had increased scores for night waking relative to children taking no medication (*p* = 0.040). There was no effect of melatonin on the night waking subscale.

### Sleep Parameters

As 32 participants had a weekly average of bedtimes later than 02:00 and/or wake up times later than 10:00 were suspected to have a delayed sleep phase disorder, they were not included in the following sleep parameter analysis. Of these 32 excluded participants, mean age was 10.71 (SD = 4.43). Average time to bed was 22:24 (SD = 2:44), average falling asleep time was 01:27 (SD = 4:54), and average morning wake-up time was 09:58 (SD = 2:47). Night waking duration was on average 113 min (SD = 99.94) and total sleep time was 7.31 h (SD = 4.13). Mean CSHQ subscale scores were as follows: bedtime resistance (M = 22.06, SD = 3.46), parasomnias (M = 9.88, SD = 2.64), sleep onset delay (M = 2.57, SD = 0.75), daytime sleepiness (16.72, SD = 3.56), sleep anxiety (M = 7.05, SD = 2.74), sleep disordered breathing (M = 3.76, SD = 1.52), sleep duration (M = 7.20, SD = 1.88), and night waking (M = 5.81, SD = 2.32).

#### Bedtime

A later bedtime (i.e., the time the child was put to bed) was significantly correlated with older age. There was a significant difference between age groups and time to bed. Pairwise comparisons revealed children aged 3–5 years old (KS0) had a significantly earlier bedtime compared to 7–11 years old (KS2), 11–14 years (KS3) (both *p* < 0.001), and 14–16 years (KS4) (*p* = 0.006). Children aged 5–7 years old (KS1) had a significantly earlier bedtime compared to 7–11 year olds (KS2) (*p* < 0.001), 11–14 years (KS3) (both *p* < 0.001), and 14–16 years (KS4) (*p* = 0.008). Moreover, children aged 2–3 years had a significantly earlier bedtime compared to children aged 11–14 years (KS3) (*p* = 0.032).

There was a significant difference between diagnosis groups for time to bed; however, after correcting for multiple comparisons, no significant group differences were found. No significant differences were found between bedtime and medication or melatonin groups.

#### Falling Asleep Time

Falling asleep time (i.e., the time the child fell asleep) was significantly correlated with older age. There was a significant difference between age groups and falling asleep time. Pairwise comparisons revealed children who were 2–3 years old had a significantly earlier falling asleep time compared to those who were 7–11 years (KS2) (*p* = 0.024), 11–14 years (KS3) (*p* = 0.004), and 14–16 years (KS4) (*p* = 0.003). In addition, 3–5 year olds (KS0) had a significantly earlier falling asleep time compared to those who were 5–7 years (KS1) (*p* = 0.024), 7–11 years (KS2), 11–14 years (KS3), and 14–16 years (KS4) (all *ps* < 0.001). Furthermore, children who were 5–7 years old had a significantly earlier falling sleep time compared to those who were 7–11 years (KS2) (*p* = 0.011), 11–14 years (KS3) (*p* = 0.004), and 14–16 years (KS4) (*p* = 0.006).

There was a significant difference between primary diagnostic group and their falling asleep time; however, after correcting for multiple comparisons, no significant group differences were found.

There was no significant difference between medication groups for falling asleep time. Mean time to sleep was significantly earlier for children taking melatonin compared to children not taking melatonin.

#### Duration of Night Wakings

Correlation analysis showed no significant association between age and night wakings. Findings indicated a significant difference between age groups on sleep diary night awaking. Pairwise comparisons showed children who were 3–5 years old (KS0) had significantly longer night wakings compared to those who were 5–7 years (KS1) (*p* = 0.001) and 7–11 years (*p* = 0.008).

No significant differences were found for night wakings between diagnosis groups; however, children with a sensory diagnosis had more night time waking minutes than children without a sensory diagnosis [*F*_(1, 374)_ = 3.94, *p* = 0.048, partial η^2^ = 0.01; *M* = 89.71, *SD* = 12.38; *M* = 64.03, *SD* = 63.95, respectively).

No significant differences were found between medication groups on night wakings. Mean waking at night time was significantly longer for children taking melatonin compared to children not taking melatonin.

#### Morning Wake-Time

Correlation analysis revealed a morning wake up time became significantly later with age, there were no significant differences between age groups. No significant differences were found between primary diagnosis, sensory diagnosis, or primary medication group, and morning wake-time. Mean morning wake up time was significantly earlier for children taking melatonin compared to children not taking melatonin.

#### Assumed Sleep Duration

Correlation analysis revealed nocturnal sleep duration was significantly shorter with age. There was a significant difference between age groups and nocturnal sleep duration. Pairwise comparisons indicated children who were 3–5 years old (KS0) had a significantly higher score than 7–11 years old (KS2) (*p* = 0.033).

No significant differences were found between primary diagnosis, sensory, primary medication or melatonin group, and assumed sleep duration

### Most Prevalant Sleep Problems Reported by Parents

Individual item analysis of problem scores on the CSHQ revealed that parents of children with differing diagnoses report similar problems. The parents reporting of the most prevalent three problems within each disorder group are reported in Table 8. Overall, the most common problems were “Child sleeps too little” (88%), “Child awakens once in the night” (75%) and “Child awakens more than once during the night” (75%), and an ANCOVA showed there were no significant differences between children with different primary diagnoses and total problem score rating of the CSHQ.

**Table 8:** Top three parent reported problems most commonly reported by primary diagnostic group.

**Primary diagnosis**	**Biggest problems for parents**
ASD	1.Q13 “Child sleeps too little” 85% 2. Q18 “Child awakens more than once” 77% 3. Q17 “Child awakens once in the night” 75%
ADHD	1.Q13 “Child sleeps too little” 94% 2. Q10 “Struggles at bedtime” 83% 3. Q17 “Child awakens once in the night” 77%
Brain Injury	1.Q13 “Child sleeps too little” 96% 2. Q4 “Child sleeps right amount” 88% 3. Q3 “Child falls asleep within 20 min” 80%
ID	1.Q13 “Child sleeps too little” 94% 2. Q17 “Child awakens once in the night” 93% 3. Q18 “Child awakens more than once in the night” 93%
Learning Difficulties/Dyslexia/Dyspraxia	1.Q11 “Needs parent in room” 80% 2. Q19 “Child talks during sleep” 73% 3. Q13 “Child sleeps too little” 71% AND Q18 “Child awakens more than once in the night 71%
Down's Syndrome	1.Q20 “Child is restless and moves a lot during sleep” 100% 2. Q13 “Child sleeps too little” 90% 3. Q17 “Child awakens once in the night” 88%
Cerebral Palsy	1.Q17 “Child awakens once in the night” 100% 2. Q5 “Child sleeps about the same amount each night” 100% 3. Q33 “Child is tired in the morning” 100%
Epilepsy	1.Q13 “Child sleeps too little” 80% 2. Q18 “Child awakens more than once in the night” 71% 3. Q4 “Child sleeps right amount” 67%

### Parental Intervention

Parents were asked what interventions had been tried prior to contacting the sleep service, the majority of parents (68%) had been present at bedtime in an attempt to improve their child’s sleep (see Table 9). Four hundred and twenty nine parents had tried at least one intervention and 195 parents had tried more than one intervention.

**Table 9:** Number of parents who have tried interventions prior to contacting the sleep service.

**Parent intervention**	***n***	***%***
Parent presence	408	67.9
Toys	16	2.7
Sleep environment	131	21.8
Story	10	1.7
Pacifier	11	1.8
Food or drink	74	12.3
TV	21	3.5
Tablet/Tech	1	0.2
No intervention	172	28.6
Total	844	–

*NB: Total not 601 as refers to all interventions parents had tried*.

Independent samples *t*-tests were conducted to compare those children who underwent a parent presence intervention to children who did not on CSHQ subscales (see Table 10 for means and standard deviations). Findings indicated mean sleep duration score was significantly higher for children receiving parent presence than children who were not. In addition, mean sleep anxiety score was significantly higher for children who were receiving parent presence than children who were not. Furthermore, mean daytime sleepiness score was significantly lower for children who were receiving parent presence as an intervention than children who were not. The other sleep disturbance subscales were non-significant.

**Table 10:** Means and standard deviations on sleep disturbances and sleep parameters for children whose parents have tried parent presence as an intervention vs. those who have not.

**Group**	**Parent presence intervention**		**No parent presence intervention**		***T*****-test main results**		
	***M***	***SD***	***M***	***SD***	***t***	***p***	***Cohen's d***
**Sleep disturbance**							
Bedtime Resistance	11.74	3.42	11.73	3.75	–	–	–
Sleep Onset Delay	2.50	0.73	2.55	0.71	–	–	–
Sleep Duration	7.31	1.61	6.91	1.82	−2.12	0.035	0.16
Sleep Anxiety	8.80	2.54	8.01	2.70	–.2.54	0.012	0.20
Night Waking	6.51	1.81	6.05	2.17	–	–	–
Parasomnias	11.32	2.69	10.78	3.34	–	–	–
SDB	4.00	1.51	4.04	1.50	–	–	–
Daytime Sleepiness	14.02	3.79	15.94	3.94	4.21	<0.001	0.52
**Sleep parameter**							
Bedtime	20:16	1:13	20:32	1:13	–	–	–
Falling asleep time	21:41	1:37	22:05	1:39	–	–	–
Morning wake time	06:54	1:15	07:07	1:21	–	–	–
Night Waking	63.53	60.39	83.90	88.04	–	–	–
Sleep duration	8.34	1.93	8.52	2.47	–	–	–

*NB: All significant t-test results reported for parent presence as an intervention group*.

## Discussion

This study aimed to use parental report to evaluate sleep disturbances and sleep patterns in children with NDC. We aimed to identify associations with age, diagnosis, and medication groups. Overall, we found that age, diagnosis, and medication were all associated with sleep disturbances and should contribute to clinical assessment of sleep disorders in this population.

### Chronological Age Associations

Several age-related associations were present; bedtime resistance, parasomnias, and night wakings all improved with age. However, sleep onset delay and daytime sleepiness both increased with age. In the sleep diary analysis, bedtime, falling asleep time, and morning wake up time, were all reported as later times as age increased. In addition, night wakings and sleep duration decreased with age.

Key stage age groups were analyzed to consider implications for education and learning. Several findings were consistent with developmental expectations of sleep displayed in families of children who are typically developing. For example, younger children being put to bed and falling asleep earlier than older children. However, several of the age groups showed little difference between the bedtimes, for example, between groups aged 3–5 and 5–7 years we found only a 5 min difference between bedtimes. Overall, although younger children had a longer sleep duration than older children, across children aged 3–14 mean nocturnal sleep duration was <9 h which is less than the recommended National Sleep guidelines for all age groups (9–11 h for children aged 3–14 years). In addition, for children aged 14–16, mean nocturnal sleep duration was <8 h which again is less than the recommended National Sleep guidelines for this age group (8–10 h for children aged 14–17). Moreover, older children (aged 14–16) also had the highest daytime sleepiness score, indicating insufficient night-time sleep.

Younger children had a higher bedtime resistance compared to older children, suggesting it was more difficult for them to settle down. Children under five, had the most wakings during the night, indicating fragmented sleep. In addition, younger children had more parasomnias than older children, suggesting a more disturbed sleep. These findings are similar to previous literature suggesting these sleep disturbances are commonly identified in pre-school aged TD children (42). Older children had the highest daytime sleepiness, revealing more daytime fatigue. Furthermore, 7–11 year-olds had the highest sleep anxiety, indicating this age had the most anxious thoughts with regard to sleep. Consequently, behavioral intervention focussing on this 7–11 year old age group and associated sleep anxiety and sleep onset may be beneficial in reducing daytime sleepiness.

### Differences Between Primary Diagnosis Groups

Significant differences between primary diagnosis groups were limited. This is likely due to this sample representing the high end of the clinical spectrum of sleep disturbances across several disability groups. Many previous studies have demonstrated profiling of sleep problems in one diagnostic group (16), however, our findings demonstrated that a range of sleep disturbances are present across diagnostic groups when looking at a clinical sample, suggesting that resources for management of sleep problems for families of children with NDCs may be beneficial when presented by sleep disturbance (e.g., sleep onset delay) in addition to information by diagnosis only. Differences between diagnosis groups were found for daytime sleepiness only. Children diagnosed with ASD, sensory disorders, cerebral palsy, and unspecified disorders had the highest levels of daytime sleepiness. Visual inspection of the means between diagnosis groups supports previous literature. For example, SDB is highly prevalent in children with DS (12), and children with DS and physical conditions both scored highly on SDB, however, statistically significant findings between groups were not present.

### Most Prevalent Sleep Problems Reported by Parents

These findings may add further context to the visual differences in means between disability groups. Overall, parents/carers reported the most common problems they experience with their child's sleep as “Child sleeps too little” and “child waking up one or more times during the night.” These findings support previous literature (43). Different diagnostic groups differed slightly. All parents reported their child sleeping too little as a common problem apart from parents of children with CP. Their child waking up was a common problem for all parents apart from parents of children diagnosed with brain damage or LD. Diagnosis-specific problems included struggles at bedtime for children with ADHD; whilst talking during sleep and needing parent in room where the most common problems for the LD/dyslexia/dyspraxia group. Parents of children with DS reported their child being restless and moving a lot during sleep. Overall, parents of children diagnosed with CP and brain damage reported fewer problems, such as “child sleep right amount” and “falls asleep within 20 min.” These problems highlighted by parents may reflect a need for more specific guidelines for children with NDCs on sleep duration and management of night waking.

### Sensory Conditions

Sensory conditions were of particular interest due to previous literature suggesting these affect sleep (40). Our findings demonstrated that children diagnosed with a primary sensory condition were higher in daytime sleepiness than children with DS. In addition, children with sensory conditions had longer night waking duration than children without sensory conditions as reported in the sleep diary. These findings suggest that consideration of co-occurring conditions, such as sensory conditions, should be considered when evaluating sleep problems in these populations (44). These conditions may frequently co-occur with the child's primary diagnosis, despite not being directly linked to it. The severity of co-occurring conditions should also be considered.

### Medication

Medication affected bedtime resistance, sleep anxiety, SDB, after controlling for age and multiple comparisons. Specifically, children using seizure control medication had lower bedtime resistance and lower anxiety than those children who were not using seizure control medication, interestingly, this same finding is not reflected in the diagnostic group comparisons for children with epilepsy. Although the effects of epilepsy on sleep have been reported (45), there is perhaps further consideration for the interaction between seizure control medication, anxiety, and sleep. Children taking GI medication had higher sleep anxiety than children not taking GI medication. GI problems and anxiety have both previously been associated with sleep disturbance (46).

Genitourinary medication group and “other medication” had higher SBD than children not taking genitourinary medication. Children on “other medication” had lower scores of SBD and increased night waking. Other medication included medication such as antibiotics and homeopathy. It is unclear from the data how long children had been taking medication vs. the presentation of the sleep problem.

### Melatonin

The analysis focused on melatonin as this is primarily prescribed for sleep problems and preliminary observational studies have supported the use of melatonin for sleep in NDCs (47). However, we found several interesting findings were present suggesting further research into the use and effectiveness of melatonin in children with NDC is warranted. Overall, the mean time to sleep was significantly earlier for children taking melatonin compared to those who were not. However, morning wake up time was also significantly earlier also suggesting that melatonin can be used to adjust sleep timings but not to increase sleep duration. Wake up time at night was significantly longer, is it possible this suggests that an earlier bedtime may not be beneficial to overall sleep quality, indicating that melatonin use may decrease sleep latency but not reduce wake after sleep onset, or alternatively, children may have already had these problems which is why melatonin was selected for treatment. Intrinsic melatonin abnormalities have been suggested in syndromes such as Angelman and Smith Magenis (48). However, without pre-treatment data the efficacy of melatonin for individual children cannot be reported in this study. Further investigation is needed into the use of melatonin and its effectiveness in sleep disturbances in this population (44). Several studies have considered when the use of medication is appropriate in NDC child populations for sleep treatment (49).

### Parental Intervention

Our second aim related to describing parental sleep management techniques (parental intervention). Parents (67.9%) had been present at bedtime in an attempt to improve their child's sleep. Child sleep duration was higher for children receiving this intervention. Sleep anxiety and parasomnia were also higher in those children who had received parental presence at bedtime, suggesting this may be a reason for selection of this particular intervention. Co-sleeping has been found to be a common problem reported by parents of children with NDCs, however, behavioral interventions have been reported as successful in addressing these commonly reported problems (49). Parental presence at bedtime may not be sustainable or beneficial for families and therefore this supports the need for parental support of alternative interventions for sleep anxiety and management of parasomnias.

### Limitations

There are several limitations to be considered. The data available provide age, diagnosis, and medication considerations, however, other relevant factors such as child sex, family socioeconomic status, cultural associations, and environmental factors were unavailable and therefore could not be considered in the analysis. The diversity of the sample is likely to represent the families who access the sleep service due to independently seeking support, although these services are available without fees to all families, the characterization of the families are unknown. The variability of the group and missing data for some CSHQ subscales meant sample sizes were small for some of the diagnostic and medication groups, which may limit generalizability of findings for these groups. These findings are parental report, and previous research has demonstrated differences between subjective reporting from parents and objective reporting of sleep (5). Providing objective assessment via actigraphy for subgroups would be beneficial for providing tailored treatment. It should be considered that a number of other parameters including age, other diagnosis, and other medications may influence some of the findings, although not all of these could be considered for each of the analysis. Given the complex nature of this population, many children will have been taking several medications for multiple conditions.

### Clinical Considerations

Compared to a typical cohort and a clinical sleep disorders population, the data from Cerebra sleep service showed that 100% of children were above the clinical threshold for overall sleep problems (see Supplementary Material for analysis). Our sample scored higher than the clinical sample of TD children with sleep disorders (36, 41), suggesting that sleep problems are more severe in children with NDC. This finding also suggests that parents are accessing the sleep service when their child's sleep is severe and early intervention may be beneficial in preventing further sleep problems developing. Similarly, sleep problems in NDC have been identified as more prevalent, severe, and persistent if left untreated (44).

The range and prevalence of sleep disturbances in the NDC population endorses use of the full CSHQ as a screening tool, in addition to a sleep diary, for evaluating sleep disorders, as previously suggested (50). The high level of clinical cut-off in the subscales relating to night time behavior indicate behavioral intervention should be beneficial in addressing some of these sleep disturbances.

Behavioral sleep interventions for children of NDCs have been generally accepted as beneficial, and are suggested as front line treatment for sleep disturbances, similar to TD populations (43). Our findings suggest focusing these behavioral interventions to improve child outcomes such as behavior, daytime fatigue, and sleep quality and anxiety, in addition to addressing parental concerns through education, such as sleep duration, which was found in this study to be parents' most common concern. It is important to consider the complexity of providing sleep education to parents of NDCs, specifically parental knowledge and the acceptance and application of principles of “sleep hygiene” (44). Overall, our findings further support the need to consider five areas outlined in Stores (44) for the treatment of sleep disturbances in NDCs, which include: intrinsic pathophysiological factors (ID, communication, and impairment of melatonin), physical comorbidities (epilepsy, SDB, pain, obesity, sensory conditions), psychiatric comorbidities (anxiety, depression), medication effects (stimulant medication), and parenting practices and associated factors (such as parental mental health, cultural factors, and socio-economic status).

## Data Availability Statement

The dataset presented in this article is not readily available due to ethical approval restrictions. Please email the corresponding author. Requests to access the datasets should be directed to Elizabeth J. Halstead, l.halstead@ucl.ac.uk.

## Ethics Statement

The studies involving human participants were reviewed and approved by UCL Institute of Education Institutional Review Board. The patients/participants provided their written informed consent to participate in this study.

## Author Contributions

EH conceptualized the study. EH and DD co-designed the study. CT, KD, and AJon contributed to the acquisition of data and data entry. AJoy and ES contributed to the design of the study. EH, AJoy, and ES contributed to the data analysis and writing the manuscript for publication. EH, AJoy, ES, and DD reviewed and edited the final manuscript. All authors have approved the final manuscript.

## Conflict of Interest

The authors declare that the research was conducted in the absence of any commercial or financial relationships that could be construed as a potential conflict of interest.
