# Supplementary material for: Sleep Disturbances and Patterns in Children With Neurodevelopmental Conditions
Source: Front Pediatr. 2021 Mar 2;9:637770. doi: 10.3389/fped.2021.637770 (PMC7961155; doi:10.3389/fped.2021.637770)
Supplement: Supplementary file 1 [file Table_1.docx]

***Supplementary Material***

**1 Clinical cut offs and comparisons.**

To place the results of the present sample in context, we compared the results on each CSHQ scale to reported data from a typical cohort and a clinical sleep disorders population previously published in Owens et al. (40). The sample was filtered to include only children aged between 4-10 years old, to match Owens et al. published data. We used one-sample *t*-tests to find that the present sample significantly differed from both the clinical and TD cohorts on all variables except the score for Parasomnias, which was not significantly different to the clinical group (marked with ‘a’ in Table 1). Every child in the present study scored above the total clinical cut-off score of 41 (*M* = 61.16, *SD* = 9.75), generated by analysis of the receiver operator characteristic curve which yielded a sensitivity of .80 and a specificity of .72 for diagnosing sleep problems (40). Moreover, for the subscale items which have a clinical cut-off validated in previous literature (40), 84.70% of the population scored over the clinical cut-off for sleep onset delay, 91.48% scored over the clinical cut-off for sleep duration, 81.05% scored over the clinical cut-off for night waking’s, and 44.00% scored over the clinical cut-off for sleep disordered breathing.

Table 1. Means and standard deviations of sleep disturbance scores across cohorts.

|  | |  | | | Owens (2000) | | | | | | | |  |  |
| --- | --- | --- | --- | --- | --- | --- | --- | --- | --- | --- | --- | --- | --- | --- |
|  | | Cerebra cohort (4-10 years) | | | Clinical cohort  (4-10 years) | | | | | TD cohort  (4-10 years) | | |  |  |
| CSHQ scores | | *M* | *SD* | *M* | | *SD* | | | *M* | | *SD* | | | |
| Total score Bedtime Resistance Sleep Onset Delay Sleep Duration Sleep Anxiety Night Waking’s  Parasomnias^a^ Sleep Disordered Breathing Daytime Sleepiness | 62.25 12.12 2.53 7.05 8.95 6.38 11.41 4.08 14.55 | | 10.27 3.42 0.73 1.68 2.44 2.03 3.05 1.52 3.92 | 68.40 9.43 1.80 4.94 7.09  5.69 11.22 4.71 11.99 | | | 13.70 3.49 0.57 1.98 2.44 1.60 2.53 2.54 3.39 | 56.20 7.06 1.25 3.41 4.89 3.51 8.11 3.24  9.64 | | | | 8.90 1.89 0.88 0.93 1.45 0.89 1.25 0.63 2.80 | |  |

^a^ Non-significant difference.

Reference

40. Owens JA, Spirito A, McGuinn M, Nobile C. Sleep habits and sleep disturbance in elementary school-aged children. J Dev Behav Pediatr. (2000) 21:27–36. doi: 10.1097/00004703-200002000-00005.
